# Polarizing intestinal epithelial cells electrically through Ror2

**DOI:** 10.1242/jcs.146357

**Published:** 2014-08-01

**Authors:** Lin Cao, Colin D. McCaig, Roderick H. Scott, Siwei Zhao, Gillian Milne, Hans Clevers, Min Zhao, Jin Pu

**Affiliations:** 1 School of Medical Sciences, Institute of Medical Sciences, University of Aberdeen, Aberdeen AB25 2ZD, UK; 2 Department of Dermatology, Department of Ophthalmology, Institute of Regenerative Cures, University of California, Davis, CA 95616, USA; 3 Department of Bioengineering, University of California, Davis, CA 95616, USA; 4 Hubrecht Institute for Developmental Biology and Stem Cell Research & University Medical Centre Utrecht, 3584 CT Utrecht, The Netherlands

**Keywords:** Cell polarization, Intestinal epithelial cells, Electric field, Transepithelial potential difference, Ror2

## Abstract

The apicobasal polarity of enterocytes determines where the brush border membrane (apical membrane) will form, but how this apical membrane faces the lumen is not well understood. The electrical signal across the epithelium could serve as a coordinating cue, orienting and polarizing enterocytes. Here, we show that applying a physiological electric field to intestinal epithelial cells, to mimic the natural electric field created by the transepithelial potential difference, polarized phosphorylation of the actin-binding protein ezrin, increased expression of intestinal alkaline phosphatase (ALPI, a differentiation marker) and remodeled the actin cytoskeleton selectively on the cathode side. In addition, an applied electric field also activated ERK1/2 and LKB1 (also known as STK11), key molecules in apical membrane formation. Disruption of the tyrosine protein kinase transmembrane receptor Ror2 suppressed activation of ERK1/2 and LKB1 significantly, and subsequently inhibited apical membrane formation in enterocytes. Our findings indicate that the endogenous electric field created by the transepithelial potential difference might act as an essential coordinating signal for apical membrane formation at a tissue level, through activation of LKB1 mediated by Ror2–ERK signaling.

## INTRODUCTION

Polarization of gut epithelial cells (enterocytes) involves the generation of the brush border membrane (BBM) or apical membrane at the luminal side of the intestine. The activation of the serine/threonine kinase LKB1 (also known as STK11) induces complete BBM formation in intestinal epithelial cells (Baas et al., 2004). To create tissue polarity, the polarity of individual cells in a sheet must be coordinated and this requires a tissue-level signal acting as a vector to specify the apical and/or basolateral surfaces of every cell. The endogenous electrical signal (transepithelial potential difference, TEP) could provide such a directional signal for chemotactic molecules and morphogen gradients. The TEP is an inherent property of transporting epithelia and arises from spatial variations in the functioning of ion pumps, channels or leak conductance across individual cells, and across layers of cells (McCaig et al., 2009). The normally basolateral Na^+^/K^+^-ATPase is one of the important molecules in the generation of the TEP (Wilson, 1997; Wilson, 2011). Across human intestine, there is a TEP of −25±7 mV, lumen negative (mean±s.e.m.). This is the equivalent of a direct current electric field of ∼500 mV/mm across the epithelial layer because the epithelium of human intestine is ∼50 µm thick (Archampong and Edmonds, 1972). Similarly sized endogenous direct current electric fields have been demonstrated in development, regeneration and pathology (Levin, 2012; Piccolino, 1998; Stern and MacKenzie, 1983). In addition, we have reported that the polarization of the Golgi complex in CHO cells is determined by an applied physiological electric field (Cao et al., 2011; Pu and Zhao, 2005). Here, we test the novel hypothesis that the TEP acts as a signal that coordinates the actin polarity of every individual epithelial cell such that, at a tissue level, the correct localization of the apical membrane arises throughout the entire intestine.

## RESULTS AND DISCUSSION

### A physiological electric field induces cathodal polarization of intestinal epithelial cells

**Fig. 1.**
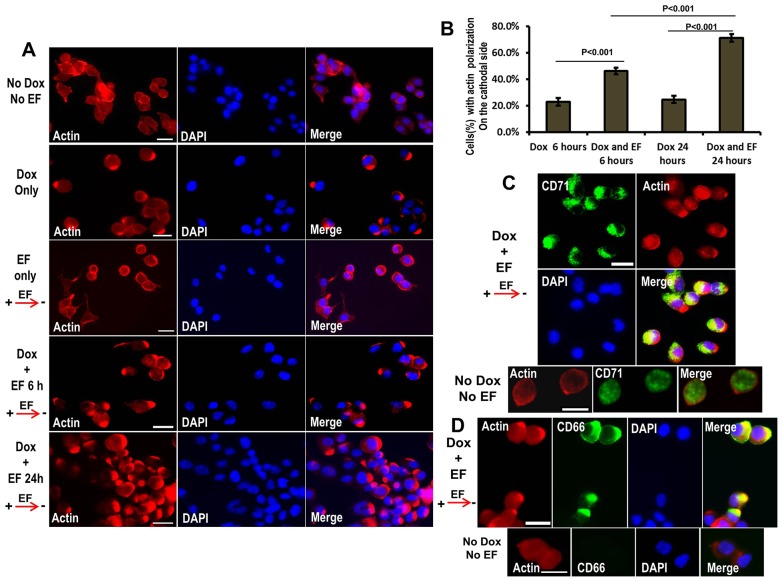
**Electrical signals polarize actin, CD66 and CD71 in enterocytes.** (A) Cells were stained with phalloidin (F-actin label indicative of apical membrane) in control cells, and cells treated with Dox only, electric field (EF) only, Dox+electric field groups. Electric field direction is shown with red arrows. Electric field stimulation causes a polarization of F-actin on the cathodal side of the cells (shown at the right). (B) Quantitative analysis of LS174T-W4 cells with actin polarization facing the cathode in an applied electric field. Analysis methods are shown in supplementary material Fig. S3. Values are mean±s.e.m. for three experiments. (C) Upper two rows: LS174T-W4 cells were treated with electric field and Dox for 24 hours. Polarized actin labeling faced the cathode, and the basal membrane marker CD71 was localized selectively at the anode side. The lower row are control cells, which show no actin or CD71 polarization. (D) Upper row: CD66 staining, as an apical marker, showing an overlapping distribution with cathodal actin polarization in LS174T-W4 cells in applied electric field plus Dox. Lower row: there is no actin and CD66 polarization in no Dox plus no electric field control cells. The applied electric field was 100–200 mV/mm. Scale bars: 10 µm.

We first mimicked the endogenous electric field established by applying a physiological electric field to cultured enterocytes to test the effects on the formation of polarization. Doxycycline (Dox)-induced LKB1 activity was required to form an apical membrane in single LS174T-W4 intestinal epithelial cells (Baas et al., 2004). When LS174T-W4 cells were treated with Dox or an applied physiological electric field plus Dox for 6 hours, 64%±3.2 and 70%±2.5 (mean±s.e.m.) of cells, respectively, showed an actin cap (apical membrane formation, supplementary material Fig. S2). In LS174T-W4 cells with an apical membrane, the actin was significantly reoriented to the cathodal-facing side in cells treated with Dox plus electric field compared to cells treated with Dox only (46.3% and 23%, *P*<0.01). When we extended the application of an electric field of 100 mV/mm plus Dox to 24 hours, the proportion of cells with apical membrane facing cathodally increased significantly to 71.2%±3.5 (*P*<0.001, compared to Dox only, 24.7%) (Fig. 1A,B). In LS174T-W4 cells, the applied electric field alone could not induce significant apical membrane formation owing to low endogenous LKB1 expression without Dox induction (supplementary material Fig. S2B). This result indicated that LKB1 is required for electric-field-induced actin polarity. Furthermore, the applied electric field not only triggered the creation of an apical membrane specifically on the cathodal side of cells but also increased the proportion of cells with the basal marker CD71 at the opposite pole, anodally (56.4% and 28% Dox only, *P*<0.001, Fig. 1C; supplementary material Fig. S4). CD66 is a member of the carcinoembryonic antigen (CEA) family and is an apical marker in epithelium polarization (Virji et al., 1996). In polarized LS174T-W4 cells, the location of CD66 overlapped quantitatively with actin polarization when cells were treated with electric field and Dox (Fig. 1D; supplementary material Fig. S3C). Finally, to determine whether electric-field-induced cell motility (galvanotaxis) might have induced cathodal assembly of some polarity markers, we measured the galvanotaxis of LS174T-W4 cells using real-time recording. We found no galvanotaxis in these cells, and so can eliminate this as a causal factor (supplementary material Movie 1).

### A physiological electric field activates LKB1 and ezrin, and increases ALPI expression

**Fig. 2.**
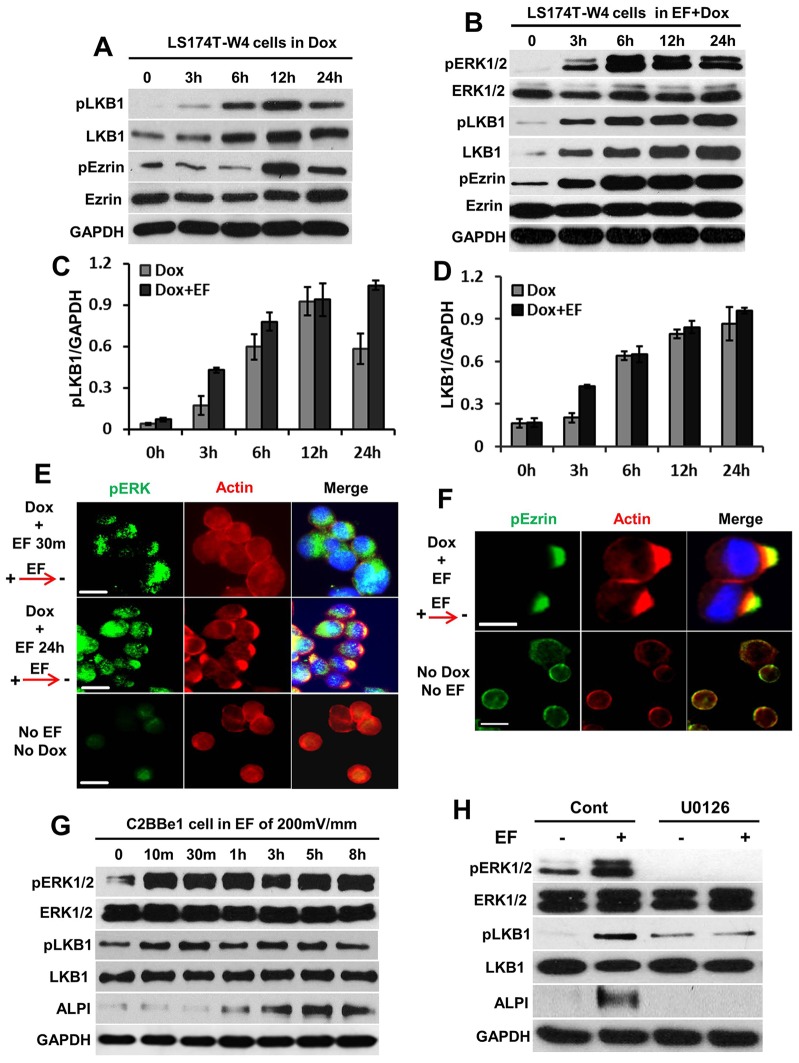
**A physiological electric field activates ERK1/2, LKB1 and ezrin and promotes expression of ALPI.** (A) Western blot analysis showing the timecourse of the increased phosphorylation of LKB1 and ezrin (pLKB and pErzin, respectively), and LKB1 expression after treatment with Dox. (B) Dox plus an electric field (EF) activated ERK1/2, LKB1 and ezrin (as shown by the increase in their phosphorylated forms) and upregulated expression of LKB1 in LS174T-W4 cells. (C,D) Quantification of pLKB1(C) and LKB1 (D) expression relative to GAPDH. Values are mean±s.e.m. for three experiments. (E,F) Actin and phosphorylated ERK1/2 (E, pERK) or phosphorylated ezrin (F) are colocalized and polarized towards the cathodal side in LS174T-W4 with Dox and electric field treatment. Specifically, as shown in the upper panel of E, a substantial amount of pERK was present at the cathode side after electric field application for 30 minutes, whereas no actin cap formation was evident at this early stage. In control cells (lower panel), actin, pEzrin and pERK accumulated uniformly around the cells. Scale bars: 10 µm. (G) The time course of electric-field-induced activation of ERK and LKB1 and enhanced ALPI expression in C2BBe1 cells. The expression of total ERK1/2 and LKB1 were not affected by electric field. (H) Pharmacological inhibition of pERK1/2 by U0126 abolished electric field-induced ALPI upregulation and impaired electric-field-activated LKB1 expression. U0126 did not affect the expression of total ERK and LKB1. GAPDH was loading control.

LKB1 is a key regulator involving establishment and maintenance of cell polarity (Baas et al., 2004; Nakano and Takashima, 2012). In LS174T-W4 cells, LKB1 started to be activated (i.e. phosphorylated LKB1 appeared) within 3 hours of Dox induction; total LKB1 expression was upregulated slightly at 3 hours and increased significantly at 6 hours of induction (Fig. 2A). However, in cells treated with Dox plus electric field, activation of LKB1 was more substantial and lasted longer (>24 hours) than that with Dox treatment alone (Fig. 2B,C). In C2BBe1 cells, endogenous LKB1 was activated by application of only an electric field within 10 minutes and remained continually activated for 8 hours (Fig. 2G). These results imply that an electric field directs apical membrane formation in intestinal epithelial cells through activation of LKB1. Although the electric-field-induced increase in the expression of LKB1 was more pronounced than with Dox alone at 3 hours in LS174-WT cells (Fig. 2A,B,D), LKB expression did not show any changes upon electric field treatment in C2BBe1 cells (Fig. 2G). We consider that this might be due to an interaction between the applied electric field and Dox when inducing exogenous LKB1 expression during the first 3 hours. Intestinal alkaline phosphatase (ALPI) is involved with both the breakdown of dietary cholesterol and the absorption of Ca^2+^ and is regarded as a functional marker of brush border membrane (apical membrane). We found that expression of ALPI was upregulated after 1 hour of electric field exposure, with continuing overexpression up to 8 hours in C2BBe1 cells (Fig. 2G). These results further confirm that the electric field appears to trigger polarization of actin in enterocytes and that this is dependent upon LKB1 activation.

Ezrin is an actin-binding protein that is concentrated on the apical microvilli of a wide variety of epithelial cells (Berryman et al., 1993). During polarization of single enterocytes, the recruitment of phosphorylated ezrin to the apical surface of epithelial cells represents an essential step in BBM biogenesis (ten Klooster et al., 2009). Here, we found that the applied electric field plus Dox activated ezrin much earlier (<3 hours, band at 80 kDa) and that this was maintained for longer (up to 24 hours) than with Dox only. Total ezrin expression was not changed with or without electric field application (Fig. 2A,B). In addition, we found that phosphorylated ezrin was almost completely within the actin cap at the cathode side of LS174T-W4 cells exposed to the electric field plus Dox, but there was no actin and ezrin cap formation in control cells (Fig. 2F). This suggests that an applied electric field not only promotes the polarization of actin, but also activates and polarizes ezrin, which is a key molecule in the polarization of intestinal epithelial cells (ten Klooster et al., 2009).

### ERK1/2 mediates electric field-induced activation of LKB1

Recent studies have identified LKB1 as a downstream target of ERK1/2 (also known as MAPK3 and MAPK1, respectively) activation in hepatic satellite cells and melanoma cells (Woodhoo et al., 2012; Zheng et al., 2009). Phosphorylation of ERK1/2 (pERK1/2) is crucial for the establishment of cell polarity in migrating cells (Bisel et al., 2008), and activation of the ERK1/2 MAPK cascade induces villin promoter activity and stimulates intestinal epithelial differentiation (Houde et al., 2001; Rieder et al., 2005). Here, we found that an applied physiological electric field induced rapid and sustained pERK1/2 in both LS174T and C2BBe1 cells (Fig. 2B,G). Furthermore, disruption of pERK1/2 with U0126 inhibited the activation of LKB1 and reduced electric-field-induced ALPI upregulation (Fig. 2H). The expression of total ERK1/2 was not affected by an electric field or by U0126. These data suggest that phosphorylation of ERK1/2 might mediate electric-field-induced LKB1 activation and ALPI expression.

ERK1/2 is localized at the apical side of the enterocyte and might act locally to modulate microvilli architecture and BBM-associated function (Boucher and Rivard, 2003). We have also previously shown that a physiological electric field induces asymmetric ERK signaling cathodally in corneal epithelial cells (Zhao et al., 2002). These findings prompted us to investigate whether electric field exposure would induce polarized ERK activation in enterocytes. We found that the physiological electric field plus Dox induced ERK1/2 phosphorylation preferentially at the cathodal side in 30 minutes, before actin cap formation was evident. Furthermore, after long term (24 hours) exposure to the electric field and Dox, activated ERK1/2 not only colocalized with actin in LS174T-W4 cells, but also throughout the whole cell (Fig. 2E). This suggests that the activated ERK1/2 induced by an electric field plays other biological functions. There was no asymmetry of activated ERK1/2 in control cells (Fig. 2E). Our findings indicate that polarized pERK1/2 may contribute to electric-field-induced cathodal actin polymerization in intestinal epithelial cells.

### Ror2 is required for electric-field-induced ERK1/2 activation and ALPI upregulation

**Fig. 3.**
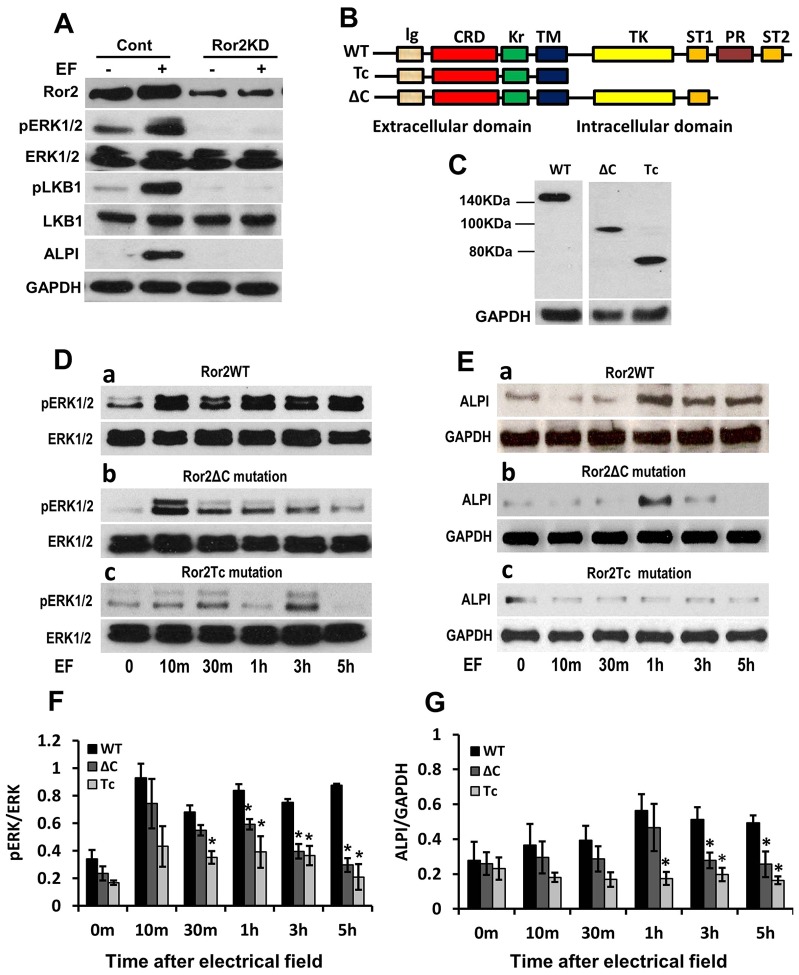
**Ror2 inhibits electric-field-induced activation of ERK1/2 and ALPI upregulation.** (A) Knockdown of Ror2 with siRNA (Ror2KD) in C2BBe1 cells abolished electric field (EF)-induced phosphorylation of ERK1/2 (pERK1/2), LKB1 (pLKB1) and expression of ALPI. However, no changes of total ERK1/2 and LKB1 were observed after knockdown of Ror2. Cont, knockdown with non-targeting siRNA. (B) Schematic constructs of the wild-type Ror2 (WT) and the two Ror2 mutants (Tc and ΔC) used in these experiments. Ig, Ig like domain; CRD, cysteine-rich domain; Kr, Wnt and a kringle domain; TM, transmembrane domain; TK, tyrosine kinase domain; ST, serine/threonine-rich domain; and PR, proline-rich domain. (C) The different size of the GFP-tagged exogenous Ror2WT, and the ΔC and Tc mutations as demonstrated by using anti-GFP antibody in western blotting. (D) ERK1/2 was immediately activated at 10 minutes in Ror2WT C2BBe1 cells and remained elevated for 8 hours in an applied electric field of 200 mV/mm (a). Electric-field-induced formation of pERK1/2 was attenuated in both Ror2 mutants (b,c). Total ERK did not change between WT and mutated Ror2 in an applied electric field. (E) An applied electric field increased ALPI expression in Ror2 WT cells within 1 hour (a), but was not able to induce marked upregulation of ALPI in both Ror2 mutants (b,c). (F,G) Quantification of pERK1/2 (F) and ALPI (G) relative to total ERK1/2 and GAPDH, respectively. Values are mean±s.e.m. *P<0.05 compared to Ror2 WT control. All results were analyzed from three independent experiments.

Ror2 is expressed in murine small intestinal epithelia along the entire crypt–villus axis and plays crucial roles in Wnt5a-induced cell migration by regulating formation of lamellipodia and reorientation of the microtubule-organizing center (MTOC) (Nomachi et al., 2008; Pacheco and Macleod, 2008). Ror2 and Vangl2 form a Wnt-induced receptor complex that is essential to establish planar cell polarity (PCP) (Gao et al., 2011). Wnt5a and Ror2 signaling also regulates villin expression through phosphorylation of ERK1/2 in intestinal epithelial cells (Cheung et al., 2011). Here, we found that inhibition of Ror2 expression with small interfering RNA (siRNA) abolished pERK1/2, pLKB1 and ALPI expression in C2BBe1 cells, but that the expression of total ERK1/2 and LKB1 was not affected (Fig. 3A). To extend these observations, we used a wild-type Ror2 (Ror2WT) and two Ror2 mutant constructs to establish stably transfected cell lines that express exogenous Ror2WT or Ror2 mutants (Ror2ΔC and Ror2Tc) tagged to GFP. Ror2ΔC and Ror2Tc have deletions in the cytoplasmic C-terminal region, containing the proline-rich domain (PRD), or in most of the cytoplasmic region of Ror2, respectively (Fig. 3B). The expression level of exogenous Ror2WT and the mutants ΔC and Tc in C2BBe1 cells was assessed by western blot (Fig. 3C). We found that the continuous activation of ERK1/2 by the applied electric field was reduced in the Ror2 mutants after 1 hour (Fig. 3D,F). Moreover, electric-field-induced upregulation of ALPI expression was reduced (ΔC) or abolished (Tc) in Ror2 mutants compared to Ror2WT (Fig. 3E,G). Collectively, the intracellular regions of Ror2, particularly the PRD, are responsible for the electric-field-induced ERK phosphorylation that mediates activation of the polarity protein LKB and polarization of enterocytes.

### The endogenous electric field might regulate apical membrane formation through Ror2, ERK1/2 and LKB1

**Fig. 4.**
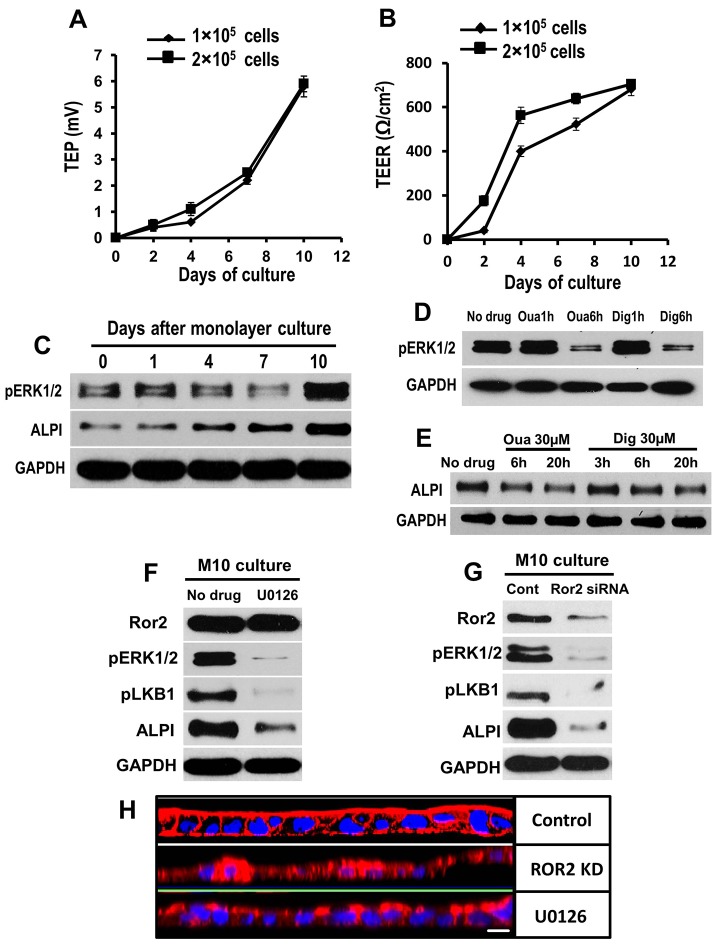
**Generation of endogenous electric field (TEP) and polarity molecules are temporally associated with apical membrane formation in C2BBe1 monolayer transwell culture.** (A,B) C2BBe1 cells form tight junctional complexes, which allow the generation of a steady time-dependent increase in TEP and TEER in insert monolayer cultures. The TEER reached a peak at ∼1 week after monolayer culture, whereas the TEP kept increasing over 10 days. Values are mean±s.e.m., *n* = 12 in each group. (C) Activation of ERK1/2 and expression of ALPI increased significantly over time in C2BBe1 monolayer cultures. (D, E) Inhibition of TEP by ouabain or digoxin effectively blocked the activation of ERK and reduced the expression of ALPI in 10-day monolayer C2BBe1 cultures. (F) The MEK inhibitor (U0126) effectively inhibited activation of ERK1/2 and LKB1, and reduced ALPI expression in 10-day monolayer (M10) cultures. No change of Ror2 expression was detected between U0126-treated and untreated samples. (G) In M10 culture, knockdown of Ror2 with siRNA markedly inhibited phosphorylation of LKB1 and ERK1/2, and downregulated expression of ALPI. (H) Confocal images (*z*-axis scanning) showed that the pronounced apical membrane architecture of C2BBe1 monolayers (upper row, staining with phalloidin–TRITC) was disrupted by suppression of Ror2 (middle row) and U0126 (pERK inhibitor) (lower row). Scale bar: 20 µm. GAPDH was used as the loading control for western blot.

The 3D epithelial culture system offers an especially relevant model because epithelial cells organize into complex structures resembling their *in vivo* architecture (O'Brien et al., 2002). After 10 days of transwell culture, C2BBe1 cells form an apical membrane in confluent monolayers and this is clearly defined by staining for actin. We also determined, using a Millicell ERS system, that a TEP of −5.9±0.3 mV (mean±s.e.m.) formed across monolayer preparations with the luminal (apical) side negative (Fig. 4A). This is equivalent to an electric field of over 200 mV/mm across the monolayer, which is ∼25 µm thick. The mechanisms of TEP generation involve activation of selective ion channels, transporters and pumps that are restricted to the apical or basolateral membranes (e.g. Na^+^/K^+^-ATPase) which create ionic gradients (Achler et al., 1989; McCaig et al., 2009; Zemelman et al., 1992). In addition, we also found that C2BBe1 monolayers developed a transepithelial electrical resistance (TEER) of 693±11 Ω/cm^2^ (mean±s.e.m.) (Fig. 4B). This level of TEER is a good indicator of strong tight junction formation and epithelial barrier function, and indicates that an endogenous electric field existed across the monolayer of C2BBe1 in transwell cultures at day 10.

In monolayer cultures, ALPI expression and activation of ERK1/2 showed a substantial time-dependent upregulation with a peak on the day 10 (Fig. 4C). This mirrors the timescale of full TEP generation. Suppressing the TEP with either ouabain or digoxin reduced the activation of ERK1/2 and ALPI expression effectively (Fig. 4D,E). Furthermore, disruption of kinase activity of MEK, or of Ror2, suppressed TEP-induced activation of ERK1/2 and LKB1 and decreased ALPI expression in monolayer cultures at day 10 (Fig. 4F,G, M10). Confocal image analysis showed that the pronounced apical membrane architecture of C2BBe1 monolayers was disrupted by suppression of Ror2 and pERK1/2 (Fig. 4H). Our results suggest that Ror2 signaling is responsible for activation of ERK1/2 and LKB1 in apical membrane formation, which is consistent with what we found with the applied electric field in a 2D cell model.

In summary, our findings that the natural bioelectrical signal across the intestinal epithelium encodes epigenetically the information required for cell and tissue level polarization add new insight into the mechanistic controls of epithelial apical-basal polarity.

## MATERIALS AND METHODS

### Cell culture and transfection

LS174T-W4 cells (supplied by Jean Paul ten Klooster, Hubrecht Institute, The Netherlands) and C2BBe1 cells (ATCC), a subclone of Caco-2 human adenocarcinoma cell line, were grown in DMEM containing standard supplements. C2BBe1 cells form a polarized monolayer with an apical membrane, morphologically comparable with that of human intestine (Peterson and Mooseker, 1992). The wild-type Ror2 construct (Ror2WT) and various Ror2 mutation constructs (ΔC and Tc) (tagged with GFP and containing a neomycin resistance gene) were kindly supplied by Michiru Nishita (Kobe, Japan) (Yoda et al., 2003). To establish stable cell lines expressing Ror2 or its mutants, C2BBe1 cells were transfected using Lipofectamine 2000 (Invitrogen) according to the manufacturer's instructions. Transfected cells were selected with G418 (1 mg/ml) and screened for Ror2 and GFP expression by western blot and immunofluorescence respectively.

### Cell treatment with applied electric field

The cells were seeded in electrotactic chambers for 16–20 hours (supplementary material Fig. S1), allowing them to settle and adhere to the base of the dish, before electric field exposure. The different strengths and times of direct current electric fields were supplied through agar–salt bridges connecting Ag/AgCl electrodes through beakers of Steinberg's solution to pools of culture medium at either side of the chamber.

### Transepithelial electrical resistance and transepithelial potential difference

1–2×10^5^ C2BBe1 cells were seeded on 24-well size cell culture inserts to form monolayers (Millipore). The inserts contain a 0.4-µm pore size polycarbonate membrane pre-coated with collagen type I. Transepithelial electrical resistance (TEER) and transepithelial potential difference (TEP) were determined using a Millicell ERS system Ohm meter (MERS00002, Millipore).

### Fluorescence microscopy and confocal imaging

The cells were stained for 2 hours with antibodies to pERM, pERK1/2 (Cell Signaling) and CD71 (BD Biosciences), respectively, and then were incubated with secondary antibodies (Life Technologies), phalloidin–TRITC (Sigma–Aldrich) and CD66–FITC (gift from Jean Paul ten Klooster) for 1 hour. Images were obtained with the Zeiss Axio Observer Z1 inverted fluorescence microscope for cell sheets and confocal 700 LSM for monolayers.

### Western blotting

Western blotting was performed as described previously (Pu and Zhao, 2005). Primary antibodies used were: anti-ALPI (NOVUS); anti-Ror2 (Abcam); anti-pERK1/2, anti-ERK anti-LKB1, anti-pLKB1 and anti-pERM (Cell Signaling); anti-GFP (Abcam); and anti-ezrin and anti-GAPDH (Santa Cruz Biotechnology). For inhibitor experiments, cells were pre-incubated with 50 µM U0126 (Cell Signaling), 30 µM ouabain or 30 µM digoxin (Sigma-Aldrich) for the time indicated.

### RNA interference in C2BBe1 Cells

A mixture of four siRNA duplexes (SMARTpools) for Ror2 was purchased from Thermo Scientific. siRNA^Ror2^ comprised: 5′-GCUCAGGCAUGGAUUACAG-3′, 5′-GCAACCGGACCAUUUAUGU-3′, 5′-CGACAGACACUGGCUACUA-3′ and 5′-GUUUGCAUGUGCCGGAAUA-3′. 5×10^4^ C2BBe1 cells were plated and the next day 100 nM of siRNA duplex was transfected using Dharmafect 1 (Thermo Scientific) according to the manufacturer's specifications. For the 10-day monolayer culture (TEP generation), C2BBe1 cells were plated into a collagen I pre-coated 12-well plate (for western blotting) or coverglass (for immunofluorescence). 100 nM of siRNA duplex were transfected on the second and sixth day of culture. After 10 days of culture, cells were stained with phalloidin–TRITC, or cell lysates were collected for western blot analysis. Non-targeting siRNA was used as a negative control for all experiments.

### Analysis of polarization

Images for actin, CD71 and CD66 staining were obtained using a Zeiss inverted fluorescence microscope (Zeiss Axiovert 100, Germany). The method for location of cell polarity markers analysis was as described previously (Pu and Zhao, 2005). The location of actin and CD66 was analyzed in five different areas: (1) 45° to 135°; (2) 135° to 225°; (3) 225° to 315°; (4) 315° to 45°; and (5) top on the cell (supplementary material Fig. S3). The positive cells, in which the marker was within the quadrant facing between 45° and 315° of the electric field direction, were scored as polarized in the electric field direction. The analysis of CD71 staining was slightly modified owing to the much wider range in which this was found than for actin and CD66 location. Here, we divided the cell into four areas: 0° to 120°, 120° to 240°, 240° to 360° and a center area (supplementary material Fig. S4). When CD71 staining was located in the 120° to 240° sector, cells were counted as positively stained on the anode side. The percentage of cells with cathodal and anodal polarization was calculated as the number of positive cells divided by total polarized cells (×100).

### Statistical analysis

A minimum of three replicates was performed and analyzed for each experiment presented. Data are presented as the mean±s.e.m. A Student's *t*-test was used to assess the significant difference. Differences were considered as statistically significant at *P*<0.05.

## Supplementary Material

Supplementary Material
