# Supplementary material for: Polarizing intestinal epithelial cells electrically through Ror2
Source: J Cell Sci. 2014 Aug 1;127(15):3233–9. doi: 10.1242/jcs.146357 (PMC4117229; doi:10.1242/jcs.146357)
Supplement: Supplementary Material [file supp_127_15_3233__index.html]

Polarizing intestinal epithelial cells electrically through Ror2 — Supplementary Material 

# Polarizing intestinal epithelial cells electrically through Ror2

## JCS146357 Supplementary Material

**Files in this Data Supplement:**

- **Supplementary Material**
