# Supplementary material for: Polarizing intestinal epithelial cells electrically through Ror2
Source: J Cell Sci. 2014 Aug 1;127(15):3233–9. doi: 10.1242/jcs.146357 (PMC4117229; doi:10.1242/jcs.146357)
Supplement: Supplementary Material [file supp_127.15.3233_JCS146357.pdf]

Supplementary materials

Polarizing intestinal epithelial cells electrically through Ror2

Lin Cao[1, 2] Colin D McCaig[1] Roderick H Scott[1] Siwei Zhao[3] Gillian Milne[1] Hans Clevers[4] Min Zhao[2]* Jin Pu[1]*

[1] School of Medical Sciences, Institute of Medical Sciences, University of Aberdeen, Aberdeen, UK

[2] Department of Dermatology, Department of Ophthalmology, Institute of Regenerative Cures, University of California, Davis, USA

[3] Department of Bioengineering, University of California, Davis, USA

[4] Hubrecht Institute for Developmental Biology and Stem Cell Research & University Medical Centre Utrecht, Netherlands

* Corresponding author: Jin Pu, School of Medical Sciences, University of Aberdeen, IMS Building, Foresterhill, Aberdeen AB25 2ZD, UK. Tel: 44 (0)1224 437532, Fax: 44(0)1224 437465, E-mail: jin.pu@abdn.ac.uk

Min Zhao, Department of Dermatology, UC Davis School of Medicine, Institute for Regenerative Cures, University of California, Davis, 2921 Stockton Blvd, Sacramento, CA 95817, USA. Tel: 1 (0)9167039381, E-mail: minzhao@ucdavis.edu

Supplementary figures and legends

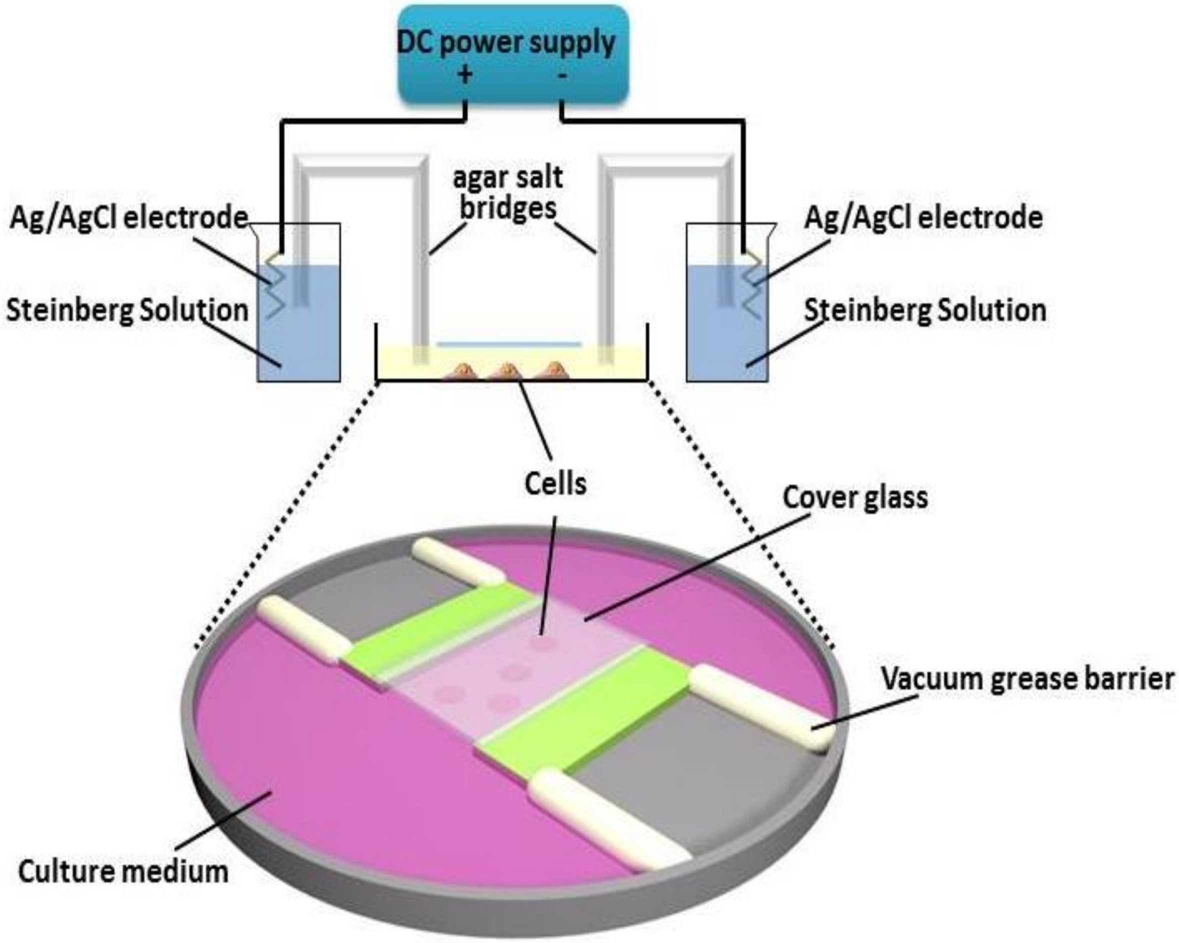

Fig. S1. The device for applying electric fields to cells to mimic the TEP. Cells were exposed to a DC electric field applied across the central chamber. The cells were cultured under the cover slide and two agar salt bridges were used to connect the culture medium with the power supply. The EF vector was parallel to the long axis of the chamber which is delineated by the two green strips of cover glass.

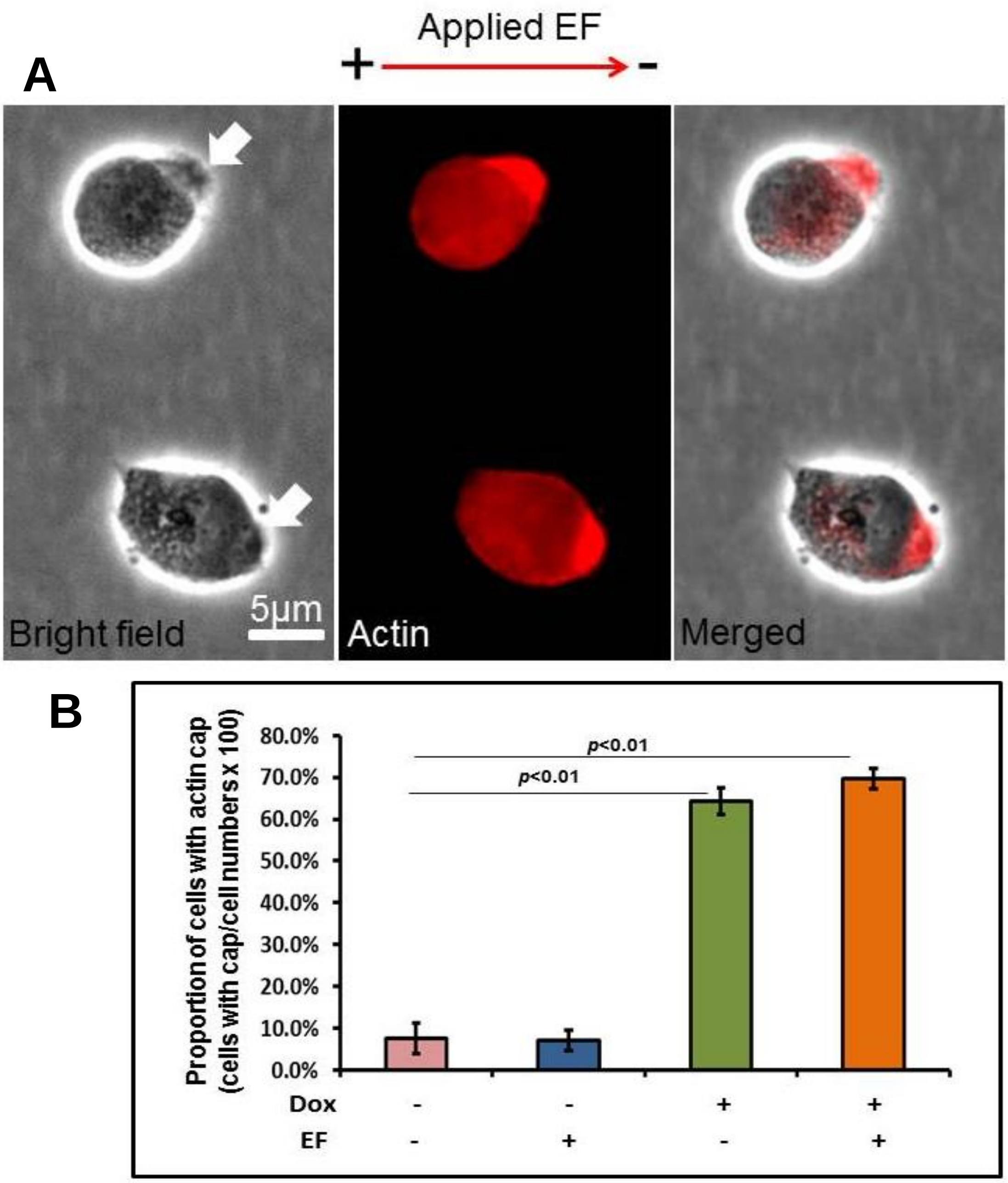

Fig. S2. Apical membrane formation in LS174T-W4 cells. (A). Cells were treated with 100mV/mm of EF and 1μg/ml Dox for 24 hours. The apical membrane (actin cap, stained with Phalloidin-TRITC) formed on the cathode-facing side of cells. A specialised protuberant membranous structure can be observed under bright field (white arrows). (B). Quantitative analysis for actin cap formation. Cells were treated with/without Dox and the applied EF. The percentage of cells with actin cap (apical membrane) is shown. Values are means ± s.e.m from three independent experiments.

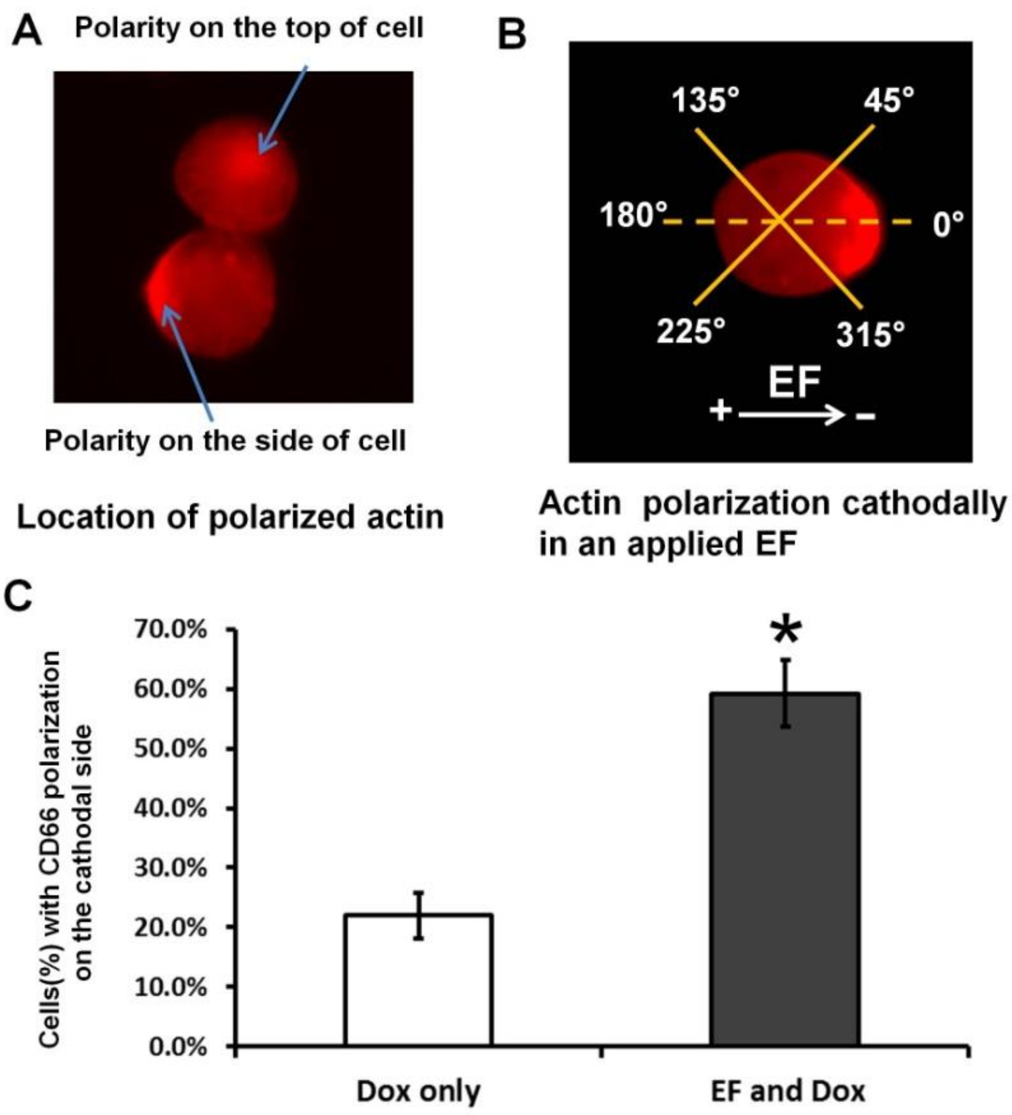

Fig. S3. Quantitative analysis for location of actin and CD66 staining in LS174T-W4 cells. (A) The actin cap is located at the margin or top of cells. (B) The location for actin or CD66 was scored as falling in to one of 5 different areas, 1) 45° to 135°; 2) 135°to 225°; 3) 225° to 315°; 4) 315° to 45°, 5) top on the cell. The cells with actin or CD66 positioned at 315° to 45° were counted as cathode/apical polarized cells. (C) Quantitative analysis of cells with CD66 cathodal polarization. The percentage of cells with cathodal CD66 polarization was calculated as the proportion of cells with CD66 on the cathode side compared to total polarized cells. Values are means ± s.e.m from three independent experiments. * $p<0.001$ compared to cells with Dox treated only.

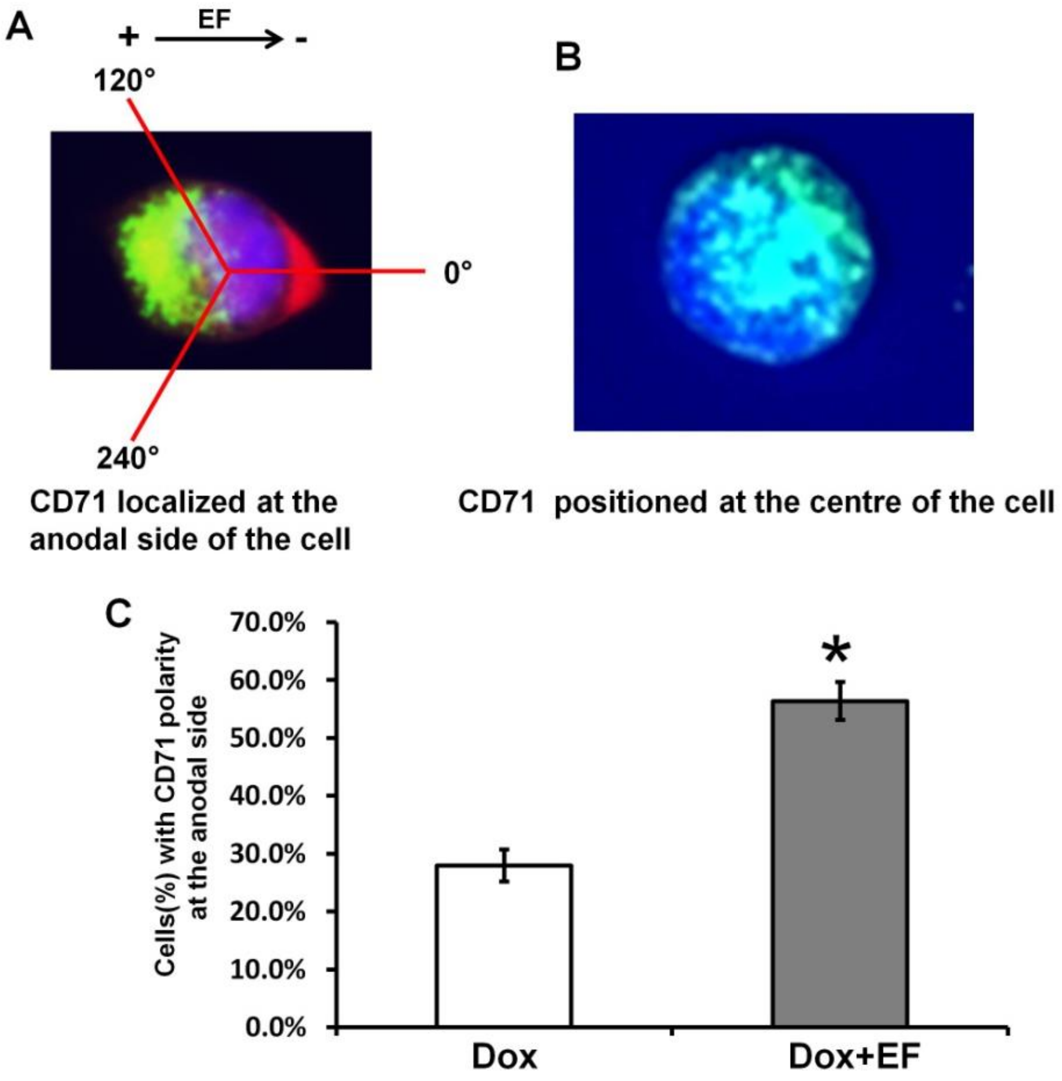

Fig. S4. Quantitative analysis for location of CD71 staining in LS174T-W4 cells. (A and B) The staining of actin and CD71 located at the margin or top of cells. The distribution of CD71 staining was assessed by dividing cells into 4 areas: 0° to 120°, 120° to 240°, 240° to 360° and centre area. The cells with the localization of CD71 at 120° to 240° were counted as anodal-positive cells. (C) Quantitative analysis of cells with CD71 anodal polarization. The percentage of cells with anodal CD71 polarization was calculated as the proportion of cells with CD71 on the anodal side. Values are means ± s.e.m from three independent experiments. * $p<0.001$ compared to cells with Dox treated only.

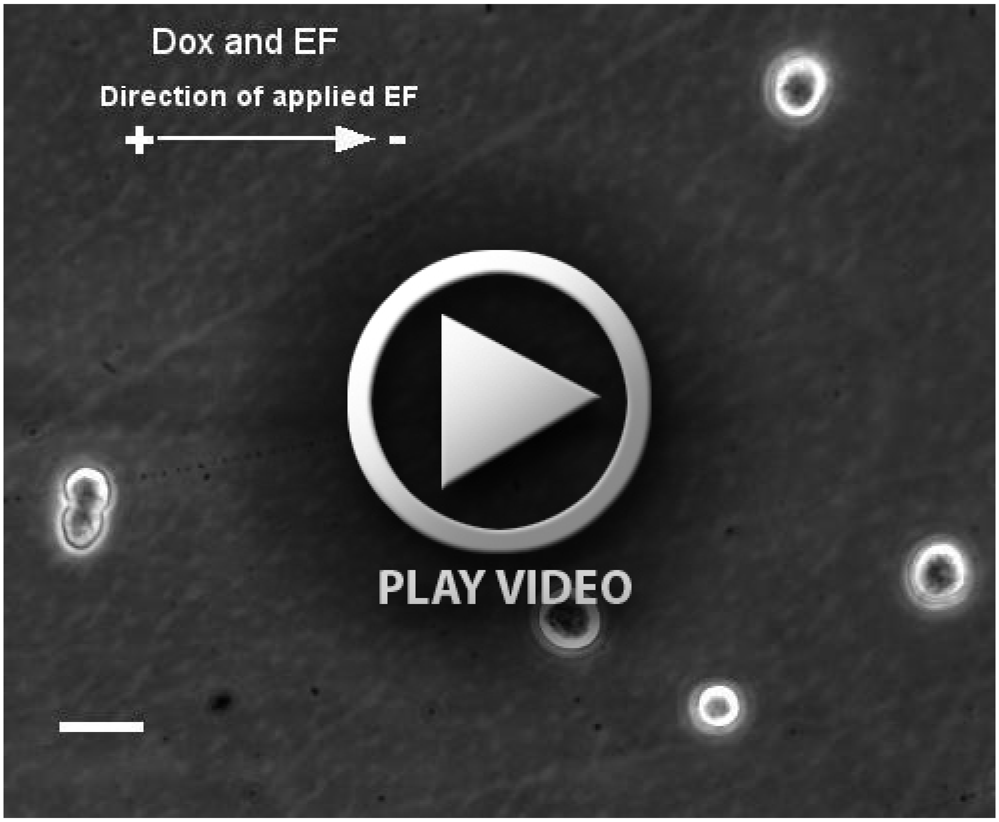

Supplemental video: galvanotaxis of LS174T-W4 cells. Cells were cultured in electrostatic chamber overnight and then were applied for 100mV/mm of electrical field plus 1µg/ml Dox. Cell images were time-lapse recorded every 10 minutes for 4 hours with a MetaMorph system in a temperature-controlled chamber. Bar= 20µm.
